# Cross-Cultural Adaptation of the Dance Functional Outcome Survey (DFOS) for Spanish Dancers

**DOI:** 10.3390/diagnostics10030169

**Published:** 2020-03-20

**Authors:** Blanca de-la-Cruz-Torres, Irene Barrera-García-Martín, Carlos Romero-Morales, Shaw Bronner

**Affiliations:** 1 Department of Physiotherapy, University of Seville, Avicena Street, 41009 Seville, Spain; bcruz@us.es (B.d.-l.-C.-T.); irebargar@gmail.com (I.B.-G.-M.); 2 Faculty of Sport Sciences, Universidad Europea de Madrid, Villaviciosa de Odón, 28670 Madrid, Spain; 3 Alvin Ailey American Dance Theater, 405 W 55th St, New York, NY 10019, USA; shaw.bronner@gmail.com; 4 ADAM Center; 322 W 52 St., #199; New York, NY 10101, USA

**Keywords:** cross-cultural adaptation, patient-reported outcome measure, dance, musculoskeletal injury, validation, physical therapy diagnosis

## Abstract

A growing number of research papers regarding Spanish-speaking dancers justifies the need for an adapted Spanish version of the Dance Functional Outcome Survey (DFOS). The objective of this study was to cross-culturally adapt and validate the DFOS for Spanish-speaking dancers. A sample of 127 healthy and injured professional and pre-professional dancers were recruited. Test-retest reliability of DFOS-Sp was examined using intraclass correlation coefficients. Construct validity compared DFOS-Sp to the Medical Outcomes Study 36-Item Short-Form Health Survey (SF-36) using Pearson correlations. Principal component analysis identified factors and internal-item consistency. Sensitivity was evaluated by generating receiver operating characteristic and area under the curve analyses. A subgroup of 51 injured dancers were followed across three time-points to examine responsiveness using repeated measures analysis of variance. Injured scores were analyzed for floor and ceiling effects. The DFOS-Sp showed high test-retest reliability (ICC2,1 ≥ 0.92). DFOS-Sp scores had moderate construct validity compared with SF-36 physical component summary scores (r ≥ 0.56). Principal component analysis (PCA) supported uni-dimensionality explaining 58% of the variance with high internal consistency (α = 0.91).Area under the curve (AUC) sensitivity values were excellent (AUC ≥ 0.82). There were significant differences across time (*p* < 0.001), demonstrating responsiveness to change, with no floor or ceiling effects. The DFOS-Sp demonstrated acceptable test-retest reliability and validity in Spanish-speaking dancers, with comparable psychometric performance to the English-language version.

## 1. Introduction

Ballet and contemporary dance are activities that require advanced levels of technical skill. Dancers are considered high-performance athletes because they perform complex, physically demanding routines and are subjected to long periods of rehearsal, similar to other elite athletes [1,2]. 

Most studies that investigate the incidence and prevalence of injuries in dance point to classical ballet as the dance modality with the highest technical demands [3,4,5]. As the foundation for other dance forms, ballet also sustains the highest rate of injuries [1,6,7]. However, both classical ballet and contemporary dancers are at high risk of low back and lower extremity injuries [7,8,9]. 

Due to the functional impact of injury on the dancer’s life, improved ways to assess and treat these challenging injuries are necessary. To analyze treatment efficacy, dance-specific outcome measures are critical to assess their unique movement skills. Bronner et al. [10] developed a self-administered questionnaire called the Dance Functional Outcome Survey (DFOS), to assess healthy state and symptom severity in injured dancers. The DFOS has been used to assess recovery following rehabilitation [11,12,13,14,15,16]. 

A growing number of research papers regarding Spanish-speaking dancers [17,18,19,20] justifies the need for an adapted Spanish version of the DFOS (DFOS-Sp). Spanish is the second most common language in the world. Clinical and research implementation of the DFOS for the Spanish-speaking population requires a systematic process of cultural adaptation and validation [21]. Our aim was to adapt the DFOS and assess its psychometric properties for Spanish-speaking dancers. Analyses included test-retest reliability, construct validity, internal consistency, sensitivity, and internal responsiveness of the DFOS-Sp in dancers with and without musculoskeletal injury to the low back or lower extremity.

## 2. Materials and Methods 

### 2.1. Instruments

The DFOS is a 14-item lower extremity and low back Likert-scale questionnaire developed upon finding ceiling effects and problems with validity when using region-specific and sport-specific measures on dancers [10]. The instrument assesses a dancer’s ability to accomplish activities of daily living (ADL) and dance-specific movements (Technique). In addition to excellent reliability and construct validity of the DFOS in adult dancers [10], research reported test-retest repeatability of the DFOS to be high in adolescent dancers [22]. 

The Medical Outcomes Study 36-Item Short-Form Health Survey (SF-36) was used as a quality-of-life outcome measure to study construct validity of the DFOS-Sp, similar to that tested in the English version [10]. The SF-36 is a self-administered generic questionnaire that yields an 8-domain profile of functional health, testing both physical and mental function. Domains include physical functioning (PF), role physical (RP), bodily pain (BP), general health perceptions (GH), vitality (VT), social functioning (SF), role emotional (RE), and mental health (MH). Each domain is scored as a z-score, with 0 representing severe disability and 100 representing no disability [23]. Standardized methods are used to calculate a Physical Component Summary (PCS), comprised primarily of PF, RP, BP, and GH-domains, and Mental Component Summary (MCS), comprised primarily of VT, SF, RE, and MH-domains. The SF-36 is reported to be suitable for use in adolescents aged 14 and older, although most studies have been conducted in adult populations [24]. The SF-36 has been translated and validated in Spanish [25].

### 2.2. Cross-Cultural Adaptation 

**Figure 1:**
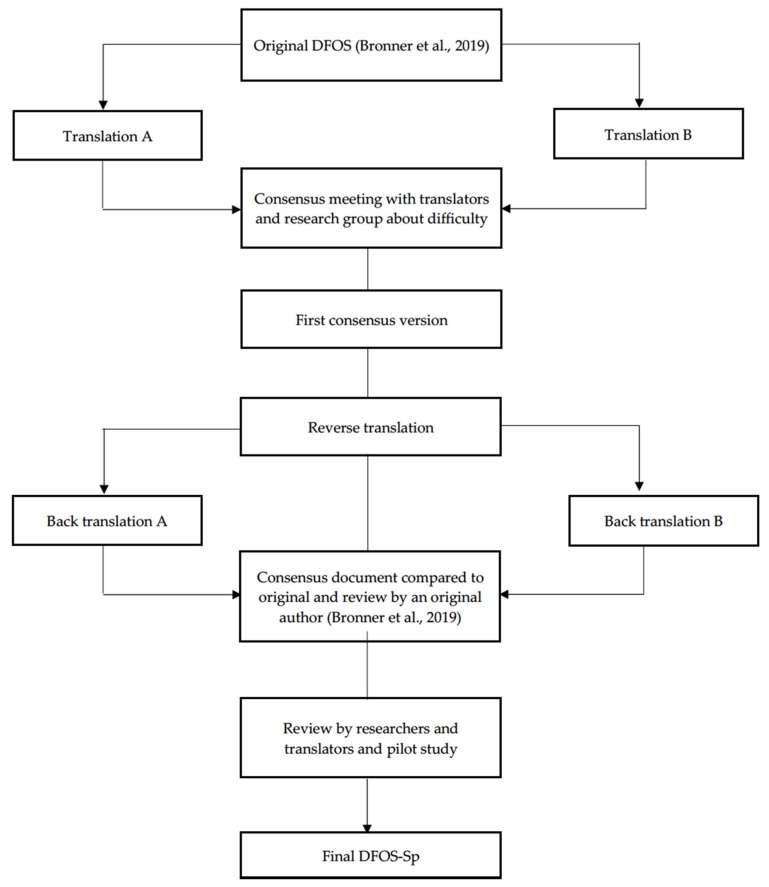
Flow chart of the steps for translation of the DFOS (English to Spanish). Abbreviations: Sp, Spanish; DFOS, Dance Functional Outcome Survey.

We followed international recommendations to perform cross-cultural adaptation and translation of the DFOS [26,27]. Cross-cultural adaptation includes cultural and linguistic adaptation of a questionnaire, and examination of its psychometric properties of reliability and validity [28]. This process consisted of six steps: (1) forward translation; (2) reconciliation; (3) back translation; (4) review and reconciliation; (5) pilot study; and (6) validation (Figure 1, Supplementary Materials survey A and B). Recommendations advise using forward translation (from source language to target language), followed by back translation to source language again, using bilingual translators who are, in one case, native speaking in the target language and in the second case, native speaking in the source language, followed by performing a thorough analysis of the new version to identify discrepancies and verify that the questionnaire will be clearly understood by study participants. In the first translation, one translators was a physical therapist and other was a sport doctor with extensive clinical experience in dance musculoskeletal disorders. In the back translation, the translators were sport physical therapists.

### 2.3. Participants 

We recruited healthy dancers from dance companies and schools and injured dancers from dance-medicine physical therapy clinics in Spain. Inclusion criterion for healthy participants included: (i) minimum of 3-years dance training including ballet and/or contemporary dance; (ii) intermediate to expert skill level; (iii) ≥15-yrs; and (iv) no low back or lower extremity injury in the previous 3-months. For injured dancers, inclusion criterion were: (i) minimum of 3-years dance training including ballet and/or contemporary dance; (ii) intermediate to expert skill level; (iii) over 15-years of age; and (iv) clinical diagnosis of any musculoskeletal injury in the low back or lower extremity verified on ultrasound or magnetic resonance imaging. Exclusion criterion included: (i) non-Spanish-speaking; (ii) pregnancy; (iii) current active disease processes; (iv) the dancers who had the presence of musculoskeletal injury in other part of the body, such as upper limb or cervical region; or (v) previous surgery. Dancers were informed of the study objectives and provided written consent according to the guidelines as approved by the local ethics committee (01/2019), which complied with all the principles set out in the Declaration of Helsinki. Parental consent was obtained if the dancer was under 18-years of age. 

After applying the inclusion/exclusion criteria, 127 dancers were recruited, comprised of 57 (45%) professional dancers and 70 (55%) pre-professional dancers (Table 1). Mean age was 20.12 ± 4.30 yrs with 11.39 ± 5.64 yrs of dance training. ‘Healthy’ dancers comprised 46% and ‘Injured’ 54% of participants. Sixty-three dancers (50%) listed ballet, 52 (41%) contemporary, while 12 (9%) listed mixed ballet/contemporary as their primary style of training. Within the 89 dancers who returned within 2-wks to answer the DFOS-Sp a second time, 48 (53%) represented ‘Healthy’ and 41 (46%) ‘Injured’ dancers, with a mean age of 18.03 ± 2.66 yrs. 

Upon enrollment, dancers answered a demographics questionnaire, DFOS-Sp and SF-36. If they took part in the reliability portion, they filled out the DFOS-Sp a second time within 7–10 days. 

A priori analysis was conducted to determine sample size for test-retest reliability and construct validity with one-group, two measurements (test-retest), effect size δ = 0.25, power = 0.95, α = 0.05, resulting in 54 subjects [29]. A sample of 89 dancers was enrolled in the test-retest and 127 dancers in the construct validity analyses. For factor analysis, a minimum of five observations per item (e.g., 70 dancers) are recommended [30]. The sample of 89 dancers were used in the factor analysis. 

To assess differences between healthy and injured groups in receiver operating characteristic (ROC) analyses, sample size estimation was conducted using a predetermined level of sensitivity of 80% (alternative hypothesis Ha= 0.80, null hypothesis H0 = 0.50, *α* = 0.05, power = 0.95) [31]. A sample of 52 per group was necessary, and 127 dancers were used in this analysis.

To assess instrument responsiveness to change, a priori analysis for sample size was conducted using one-group repeated measures ANOVA over three time-points: injured at intake (Intake Time), discharge (Discharged), and 3-months follow-up (3-months). With a small effect size δ = 0.25, power = 0.95, *α* = 0.05, a total sample of 43 was required. A sample of 52 injured dancers was used in this analysis.

### 2.4. Data Analysis

Incomplete questionnaires missing more than two items were eliminated. DFOS-Sp total, ADL and Technique scores were obtained by summing individual questions. SF-36 scores for the 8-domains and composite MCS and PCS scores were obtained using standard procedures [23]. 

Test-retest reliability analysis compared ‘Combined’, ‘Healthy’ and ‘Injured’ groups for DFOS-Sp total, ADL and Technique scores using intraclass correlation coefficients (ICC_2,1_) and related 95% confidence intervals (95% CI) (SPSS v.23, IBM Corp, Armonk, NY, USA). 

ICC values were considered low ≤ 0.49, moderate 0.50–0.69, high 0.70–0.89, and very high 0.90–1.00 [32]. For test-retest, we hypothesized high correlations (ICC ≥ 0.70). Absolute reliability, defined as variability of scores from measurement to measurement reflecting measurement *accuracy*, was determined using standard error of measurement (SEM) [33]. 

To determine construct validity of DFOS-Sp vs. SF-36, DFOS-Sp total, subscores and items were compared to SF-36 PCS, MCS, and domains using Pearson correlation coefficients (95%CI) in SPSS. We hypothesized convergent correlations between DFOS-Sp and SF-36 PCS (Pearson r ≥ 0.50) and divergent correlations between DFOS-Sp and SF-36 MCS (Pearson r ≤ 0.49), with correlation strength interpreted as weak (≤ 0.49), moderate (0.50–0.69), or strong (0.70–1.00) [34]. 

Principal component analysis (PCA) was conducted using Eigenvalues and factor loading patterns to identify and extract factors and determine Cronbach’s α (JASP v.0.9.2.0, University of Amsterdam, The Netherlands). We hypothesized a single-factor model with item correlations ≥ 0.70 and Cronbach’s α ≥ 0.70.

To conduct sensitivity analyses in the healthy group and injured groups, we conducted a *t* test for equal variances not assumed, due to unequal sample sizes (Healthy = 74; Injured = 69) and significant Levene test (*p* < 0.004). Predictive accuracy or sensitivity was measured by generating ROC curves, area under the curves (AUC), and associated 95%CI for DFOS-Sp (total, ADL and Technique subscores) in SPSS. ROC curves used DFOS-Sp scores as binary state outcome variables coded as 0 = healthy and 1 = injured. Sensitivity and specificity for cutoff values were determined. 

To determine internal responsiveness, we examined differences in DFOS-Sp and SF-36 scores in injured dancers across three time-points using repeated measures analysis of variance in SPSS. For all analyses, Mauchly’s test was used to assess assumption of sphericity. In the case of significance, the Huynh-Feldt correction was applied to the degrees of freedom and F value if the epsilon value was 0.75 or greater, and the Greenhouse-Geisser correction if epsilon was <0.75. In these cases, epsilon and corrected values (e.g., degrees of freedom, F values) are reported. Pairwise comparisons were conducted where there was a significant main effect. We hypothesized pairwise differences across time-points. 

Internal responsiveness was further defined in four ways: SEM, minimal detectable change at 95%CI (MDC_95_), standardized response mean (SRM), and effect size, using the following equations: MDC_95_ = 1.96*√2 *SEM; SRM = mean change in score/SD of change scores; effect size = mean change scores/SD of baseline scores. SEM, MDC_95_, SRM, and effect size were calculated for DFOS-Sp total and subscores and for SF-36 PCS and MCS. We anticipated SRM values, demonstrating high responsiveness [35], and large effect sizes of 0.80 or greater [36]. 

For floor and ceiling effects, we determined the percentage of dancers who achieved the highest and lowest DFOS-Sp scores within the ‘Injured’ group. Ceiling and floor effects of <15% of respondents scoring the highest or lowest scores were considered acceptable [37]. 

## 3. Results

For ‘Combined’ groups, test-retest reliability of DFOS-Sp total, ADL, and Technique scores were very high (ICC_2,1_ ≥ 0.98) (Table 2). At the item-level, reliability was also very high (ICC_2,1_ ≥ 0.92). SEM values were 1.60 (DFOS-Sp total), 0.92 (ADL), 1.02 (Technique) respectively. 

Healthy group test-retest reliability of DFOS-Sp total, ADL, and Technique scores were high (ICC_2,1_ = 0.92, 0.89, and 0.91 respectively). DFOS-Sp item correlations were high, ranging from ICC_2,1_ = 0.70–0.92, with the exception of *stairs*, *developpé*, and *rond de jambe* which were moderate (ICC_2,1_ = 0.66–0.69). SEM ranged from 1.29–1.93. ‘Injured’ group reliability scores were very high for all DFOS-Sp scores and item-level (ICC_2,1_ = 0.99). SEM ranged from 0.66–1.49. 

The combined (Healthy and Injured) pool of 127 dancers was used in analyses of construct validity. Moderate Pearson correlations were found between SF-36 PCS v. DFOS-Sp total (*r* = 0.61), and subscores (ADL *r* = 0.56 and Technique *r* = 0.58) (Table 3). Individual ADL-items were compared to PCS-domains (*Physical Function*, *Role Physical*, *Bodily Pain*) with correlations ranging from *r* = 0.26–0.54. Individual Technique-items were compared to PCS-domain *Physical Function* with correlations ranging from *r* = 0.20–0.50. In contrast, weak to no correlations were found for SF-36 MCS v. DFOS-Sp total and subscores. Individual ADL-items compared to MCS-domains (*Vitality, Social Functioning, Mental Health, Role Emotional*) and Technique-items were compared to MCS-domain *Social Functioning* were also weak. 

Data from 89 participants were used in PCA and Cronbach’s α internal consistency analyses. PCA, used single-factor loading with oblique oblimin rotation and suppression of coefficients < 0.40 [38]. Kaiser-Meyer-Olkin = 0.86 indicated sampling adequacy and Bartlett’s Test of Sphericity was significant (*χ*^2^ = 1053.222, df = 91, *p* < 0.001). Inter-item correlations loaded from 0.50–0.85 (Table 4), accounting for 58% of the variance explained and Eigenvalue = 8.12. Cronbach’s α values were high for all 14-items (α = 0.91, CI_95_ = 0.88–0.94), the 6-items within ADL (α = 0.91), and the 8-items within Technique (α = 0.91).

Data from 127 participants were used in sensitivity analyses. Significant differences were found between ‘Healthy’ (83.34 ± 7.66) and ‘Injured’ (71.35 ± 13.84) dancers for DFOS-Sp total t(104.47) = 6.348, *p* < 0.001, ADL ‘Healthy’ 36.46 ± 3.97, ‘Injured’ 31.06 ± 5.86, t(118.49) = 6.411, *p* < 0.001 and Technique subscores ‘Healthy’ 46.88 ± 4.60, ‘Injured’ 40.49 ± 8.98, t(99.79) = 5.461, *p* < 0.001. There were also differences between groups for SF-36 PCS ‘Healthy’ 55.66 ± 6.66, ‘Injured’ 44.51 ± 9.66, t(119.69) = 7.983, *p* < 0.001 and MCS PCS ‘Healthy’ 37.23 ± 5.92, ‘Injured’ 40.94 ± 9.97 t(109.064) = −2.680, *p* = 0.009.

**Figure 2:**
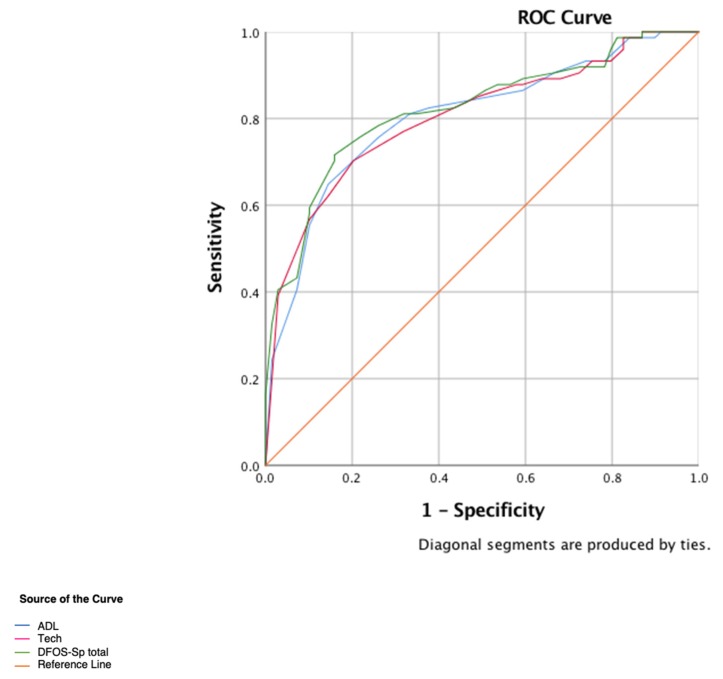
Receiver Operating Characteristic (ROC) curve for DFOS-Sp total, ADL and Technique scores. Abbreviations: ADL, activities of daily living; Tech, Technique; DFOS-Sp total, Spanish Dance Functional Outcome Survey total.

ROC curves resulted in AUC values of 0.82 (CI_95_ = 0.75–0.89) for DFOS-Sp total scores, and 0.80 (CI_95_ = 0.73–0.88) for ADL and Technique, suggesting an acceptable level of accuracy (Figure 2). Cut-offs were 80.5 for DFOS-Sp total (sensitivity and specificity values 0.76 and 0.78); 34.5 for ADL (sensitivity and specificity values 0.76 and 0.74); and 45.5 for Technique scores (sensitivity and specificity values 0.77 and 0.68) respectively. 

**Figure 3:**
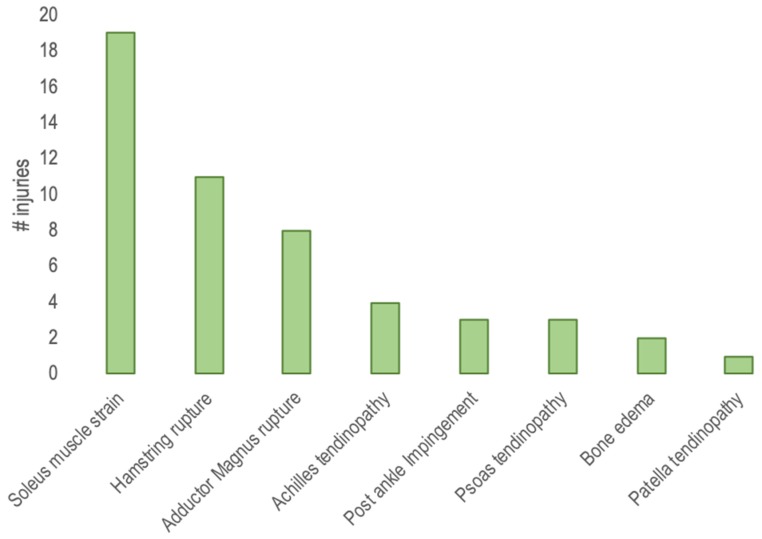
Injuries categorized by diagnosis.

Fifty-one dancers (46 female, 5 male; mean ± SD age, 19.12 ± 3.02 years) participated in internal responsiveness investigations. The dancers were recruited from a private clinic that is specialized in dance injuries. The principal investigator gave the instructions, educated the physiotherapists, and supervised the physiotherapy treatments to ensure that the procedures were conducted a standard way. The dancer’s information was given to the researchers. The main injuries were: soleus muscle strain, hamstring rupture, adductor magnus rupture, Achilles tendinopathy, post ankle impingement, psoas tendinopathy, bone edema, and patella tendinopathy (Figure 3). Treatment provided to the injured dancers included rest, eccentric exercises, ultrasound-guided percutaneous needle electrolysis, ultrasound-guided percutaneous neuromodulation, manual therapy, magnetotherapy, and activity modifications.

**Figure 4:**
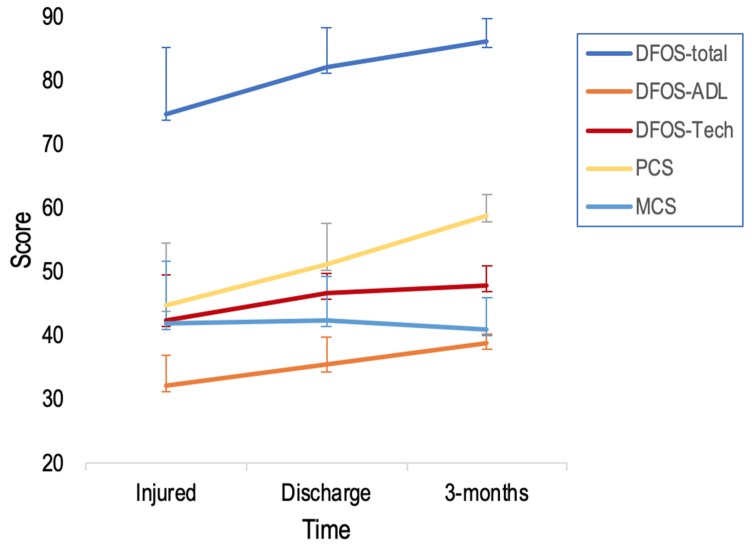
Mean ±SD DFOS-Sp and SF-36 scores across three time-points. Abbreviations: ADL, activities of daily living; DFOS, Dance Functional Outcome Survey; PCS, physical component summary; MCS, mental component summary.

Mauchaly’s Test of Sphericity was not significant; therefore sphericity was not violated. There were significant differences across time for DFOS-Sp total F(1,49) = 64.145, *p* < 0.001, ADL F(1,49) = 90.954, *p* < 0.001 and Technique F(1,49) = 28.081, *p* < 0.001 (Figure 4, Table 5). Pairwise comparisons were also significant between Injured, Discharged, and 3-months (*p* < 0.001) for DFOS-Sp total, ADL, and Technique scores. There were also significant differences across time for SF-36 PCS F(1,49) = 135.892, *p* < 0.001 but not for MCS. PCS pairwise comparisons were significant between each of the three time-points (*p* < 0.001). 

For DFOS-Sp and SF-36 scores over the three time-points, SEM values were higher at Intake Time and lowest at 3-months. MCS_95_ displayed a pattern of decreasing from high at Intake Time to low at 3-months. SRM values for DFOS-Sp and PCS scores ranged from 0.65 to 1.37 when comparing Intake Time to Discharged.

‘Injured’ group DFOS-Sp scores were examined for floor and ceiling effects. One percent of ‘Injured’ individuals had minimum DFOS-Sp total scores, and none had maximal scores. Therefore, no ceiling or floor effects were considered to be present. 

## 4. Discussion

Our main finding of this study was that DFOS-Sp items were equivalent to those in the original version, as determined by bilingual and clinical experts involved in the study. This adaptation showed good psychometric properties in Spanish dancers. The hypotheses regarding high test-retest and equivalence reliability, high internal-item consistency, and convergent and divergent correlations between DFOS-Sp and SF-36 PCS and MCS were supported. Most items in the single-factor PCA model were highly correlated. No ceiling or floor effects were considered to be present. Each finding is discussed below.

The DFOS contains French ballet terms used in ballet training around the world, simplifying translation. All dancers participating in the DFOS-Sp had ballet training. Although Beaton et al. [25] suggested that one forward translator have no knowledge of the concepts being quantified, we used bilingual native Spanish-speaking translators with dance backgrounds for the forward translation for this reason. Therefore meaning and intent were simplified. The questionnaire seemed to be easily understood by participants, who required less than 5-min to complete it independently. All participants completed the full DFOS-Sp, resulting in a maximum response rate. 

We approached our assessment of the psychometric properties of the DFOS-Sp using criteria described by the Consensus-based Standards for the selection of health Measurement Instruments (COSMIN consensus) [39]. Important measurement properties to assess in health status questionnaires include internal consistency, reproducibility, construct validity, floor and ceiling effects, responsiveness, and interpretability. These are addressed below.

Test-retest reliability correlations were very high for DFOS-Sp total and subscores for ‘Combined’, ‘Healthy’, and ‘Injured’ groups, supporting our hypothesis of ICC ≥ 0.70. These findings are similar to those reported for the original DFOS [10]. Individual-items were also very high with several exceptions in the ‘Healthy’ group. Stairs, developpé, and rond de jambe were moderately correlated. This inter-item discrepancy was also reported for rond de jambe in the ‘Healthy’ group in the English version DFOS. It is often difficult for healthy dancers to discriminate between qualitative fluctuations of ability to perform, muscle soreness, and injury pain. Because they push themselves, we often see them, for example, avoiding stairs at the end of the day due to a heavy rehearsal or performance, without being injured. 

We calculated SEM for ‘Combined’, ‘Healthy’ and ‘Injured’ groups, with similar results across the groups. SEM for DFOS-Sp was slightly lower than that reported for the English-DFOS [10] and are lower than reported values for other orthopaedic outcomes tools [40,41,42].

Construct validity was assessed by examining correlations between DFOS-Sp and SF-36. It was expected that PCS and its domains scores would have stronger correlations with DFOS-Sp, indicating convergent validity, while MCS and its domains scores would have weak or no correlation with DFOS-Sp, indicating divergent validity. Similar to previous studies, there were a moderate correlations for DFOS-Sp v. PCS and weak for DFOS-Sp v. MCS [42,43,44,45]. 

PCA found that all DFOS-Sp 14-items loaded onto a single factor. Factor-items were ≥0.74 with the exception of *overall activity* and *kneeling*. These exceeded r ≥ 0.50, so were not considered for elimination. High Cronbach’s α indicated excellent internal consistency, similar to the original DFOS [10]. Bronner et al. [10] reported that all 14-items loaded onto a single factor, indicating a single dimension in the original DFOS. However, they suggested that for the clinician to interpret the impact of injury on ADL versus Technique and make clinical decisions in rehabilitation progression, clinicians should calculate both subscores as well as the total score.

ROC analyses are used in clinical epidemiology to quantify how accurately medical diagnostic tests can distinguish between differing patient states, in this case, ‘Healthy’ and ‘Injured’ [46]. ROC plots sensitivity against 1-specificity across the full range of values. AUC assesses overall diagnostic accuracy, or discrimination, by summarizing the entire location of the ROC curve rather than depending on a specific operating point. DFOS-Sp total and subscores accurately demonstrated discrimination between healthy and injured dancers. An AUC value of 0.82, found in DFOS-Sp total, is considered excellent [47]. 

The majority of dance injuries were to the lower extremity, with most at the foot and ankle [6,7]. Injuries were typical of those seen in ballet, with 55% involving the calf, ankle, or foot.

All DFOS-Sp scores and PCS improved from Injured to Discharged and Discharged to 3-months as hypothesized. Greatest decreases in SEM and MDC_95_ values occurred from Injured to Discharge but decreased further at 3-months. This is similar to previous reports of the English-DFOS and other instruments [10,42,48,49]. SRM values demonstrated high responsiveness [35], and high effect sizes as hypothesized. SRM and effect sizes were insubstantial in MCS scores.

Floor or ceiling effects were considered if more than 15% of the ‘Injured’ group achieved highest or lowest scores [37]. Floor or ceiling effects suggest limited content validity. If a patient achieves either a maximal or minimal score and subsequently becomes better or worse, the questionnaire cannot reflect this change and its responsiveness is compromised. None of the ‘Injured’ individuals had maximum or minimum scores. Therefore, no ceiling or floor effects were present.

All dancers were Caucasian, which may limit generalizability of these findings. However, the ballet world in Spain remains primarily Caucasian which limited our population diversity. We recruited individuals from different cities in Spain to minimize bias due to cultural, semantic, or demographic factors. The DFOS-Sp has not been tested on Central or South American Spanish-speaking dancers and may require further study. In addition, future research should develop dance questionnaires, focused on the upper limb and spinal injuries as well as their impact on performance.

## 5. Conclusions

DFOS-Sp demonstrated excellent reliability, construct validity, and internal consistency for use with Spanish-speaking adult and adolescent ballet and contemporary dancers. Therefore, the DFOS-Sp is a useful tool to monitor both healthy state and functional limitation following lower extremity or back injury in ballet and contemporary dancers. 

The sample of this study consisted primarily of Spanish Caucasian participants, which may limit the generalization of the results. Central and South American Spanish may require further testing. This research supports further translation of DFOS into other languages and the adoption of it as an international reference outcome measure in prospective studies on dancers.

## Figures and Tables

**Table 1:** Demographics.

	Male	Female	Total
Subjects (%)	31 (24%)	96 (76%)	127 (100%)
Professional dancers (%)	17 (13%)	40 (32%)	57 (45%)
Pre-professional Student (%)	14 (11%)	56 (44%)	70 (55%)
Age (yrs)	21.03 ± 4.69	19.82 ± 4.14	20.12 ± 4.30
Dance Training (yrs)	10.90 ± 4.95	11.55 ± 5.86	11.39 ± 5.64
Dance training			
Ballet	18 (14%)	45 (36%)	63 (50%)
Contemporary	13 (10%)	39 (31%)	52 (41%)
Contemporary/Ballet	0 (0%)	12 (9%)	12 (9%)
Professional experience (yrs)	5.00 ± 4.23	4.83 ± 3.40	4.89 ± 3.63
Status (%)			
Healthy	10 (8%)	49 (38%)	59 (46%)
Injured	21 (17%)	47 (37%)	68 (54%)

Note: all percentages are given as % of the total. Abbreviations: Percent, %; Yrs, years.

**Table 2:** Test-retest reliability of DFOS-Sp.

Group	Combined		Healthy		Injured	
	ICC (95% CI)	SEM	ICC (95% CI)	SEM	ICC (95% CI)	SEM
**DFOS Total**	0.99 (0.98–0.99)	1.60	0.92 (0.87–0.96)	1.93	0.99 (0.99–0.99)	1.49
**ADL**	0.98 (0.97–0.99)	0.92	0.89 (0.80–0.94)	1.29	0.99 (0.97–0.99)	0.66	
Overall Activity	0.98 (0.97–0.99)		0.71 (0.48–0.84)		0.99 (0.99–0.99)	
Movement Quality	0.96 (0.95–0.98)		0.84 (0.72–0.91)		0.99 (0.99–0.99)	
Walking	0.94 (0.91–0.96)		0.82 (0.68–0.90)		0.99 (0.99–0.99)	
Stairs	0.97 (0.95–0.98)		0.66 (0.39–0.81)		0.99 (0.99–0.99)	
Stability/Symptoms	0.93 (0.89–0.95)		0.81 (0.66–0.89)		0.99 (0.99–0.99)	
Pain	0.92 (0.88–0.95)		0.77 (0.59–0.87)		0.99 (0.98–0.99)	
**Technique**	0.98 (0.98–0.99)	1.02	0.91 (0.84–0.95)	1.40	0.99 (0.97–0.99)	0.97
Plié	0.98 (0.97–0.99)		0.70 (0.47–0.83)		0.99 (0.99–0.99)	
Developpé	0.95 (0.93–0.97)		0.68 (0.44–0.82)		0.99 (0.99–0.99)	
Relevé Balance	0.98 (0.97–0.99)		0.79 (0.63–0.89)		0.99 (0.99–0.99)	
Rond de Jambe	0.93 (0.90–0.96)		0.69 (0.45–0.83)		0.99 (0.99–0.99)	
Kneeling	0.98 (0.97–0.99)		0.92 (0.86–0.95)		0.99 (0.99–0.99)	
Turning	0.97 (0.96–0.98)		0.86 (0.73–0.91)		0.99 (0.99–0.99)	
Jumping	0.96 (0.95–0.98)		0.79 (0.62–0.88)		0.99 (0.99–0.99)	
Grand Allegro	0.97 (0.96–0.98)		0.86 (0.75–0.92)		0.99 (0.99–0.99)	

Abbreviations: ICC, Intraclass Correlation Coefficient; CI, Confidence Interval; SEM, Standard error of measurement; ADL, Activities of Daily Living. Note: All correlations significant *p* < 0.001.

**Table 3:** Validity Analysis of DFOS-Sp versus SF-36.

Score	PCS	R (95% CI)	MCS	r (95% CI)
**DFOS Total**	PCS**	0.61 (0.49–0.71)	**MCS**	−0.26 (−0.42– −0.09)
**ADL**	PCS**	0.56 (0.43–0.67)	**MCS**	−0.24 (−0.40– −0.07)
Overall Activity	PF**	0.30 (0.13–0.45)	VT	0.05 (−0.12–0.22)
Overall Activity	RP**	0.26 (0.09–0.42)	SF	0.03 (−0.14–0.20)
Movement Quality	PF**	0.52 (0.38–0.64)	MH*	0.07 (−0.11–0.24)
Movement Quality	RP**	0.48 (0.33–0.60)	SF*	0.07 (−0.11–0.24)
Walking	PF**	0.48 (0.33–0.60)	SF*	0.22 (0.05–0.38)
Stairs	PF**	0.53 (0.39–0.64)	SF*	0.09 (−0.09–0.26)
Stability	BP**	0.44 (0.29–0.57)	SF*	0.14 (−0.04–0.31)
Pain	BP**	0.54 (0.40–0.65)	VT	−0.03 (−0.21– −0.14)
Pain			RE	0.11 (−0.07–0.28)
Pain			SF*	0.11 (−0.07–0.28)
**Technique**	PCS**	0.58 (0.55–0.68)	**MCS**	−0.24 (−0.40– −0.07)
Plié	PF**	0.50 (0.36–0.62)	SF	0.09 (−0.09–0.26)
Developpé	PF**	0.47 (0.32–0.60)	SF*	0.04 (−0.14–0.21)
Relevé Balance	PF**	0.38 (0.22–0.52)	SF	−0.01 (−0.19–0.16)
Rond de Jambe	PF	0.20 (0.03–0.36)	SF	−0.03 (−0.20–0.15)
Kneeling	PF**	0.48 (0.33–0.60)	SF	0.08 (−0.10–0.25)
Turning	PF**	0.31 (0.14–0.46)	SF*	0.04 (−0.14–0.21)
Jumping	PF**	0.45 (0.30–0.58)	SF*	0.19 (0.01–0.35)
Grand Allegro	PF**	0.45 (0.30–0.58)	SF	0.04 (−0.14–0.21)

** PCS correlations significant at *p* ≤ 0.026. * MCS subscores MH, SF *p* ≤ 0.037. Abbreviations: PCS, Physical Component Summary; ICC, Intraclass Correlation Coefficient; CI, Confidence Interval; MCS, Mental Component Summary; ADL, Activities of Daily Living; PF, Physical Function; RP, Role physical; BP, Bodily Pain; VT, Vitality; SF, Social functioning; MH, Mental health; RE, Role emotional.

**Table 4:** Factor Loadings.

Item Content	Factor 1
Overall Activity	0.62
Movement Quality	0.82
Walking	0.77
Stairs	0.79
Stability/Symptoms	0.77
Pain	0.76
Plié	0.81
Developpé	0.74
Relevé Balance	0.83
Rond de Jambe	0.75
Kneeling	0.50
Turning	0.85
Jumping	0.79
Grand Allegro	0.79

**Table 5:** Responsiveness of DFOS-Sp and SF-36.

Time	DFOS-Sp ADL	DFOS-Sp Technique	DFOS-Sp Total	PCS	MCS
Intake Time					
Score (Mean ±SD)	32.24 ± 4.76	42.47 ± 7.06	74.71 ± 10.48	44.81 ± 9.65	41.99 ± 9.57
SEM	0.67	1.00	1.05	1.37	1.35
MDC_95_	1.86	2.77	2.91	3.78	3.75
**Discharged**					
Score (Mean ±SD)	35.43 ± 4.46	46.65 ± 3.06	82.08 ± 6.21	51.16 ± 6.57	42.46 ± 6.72
SEM	0.63	0.43	0.62	0.93	0.95
MDC_95_	1.75	1.20	1.72	2.58	2.64
Δ (Injured minus Discharged)	3.20	4.18	7.37	6.35	0.47
SRM	0.82	0.65	0.78	1.37	0.12
ES	0.67	0.59	0.70	0.66	0.05
**3-months**					
Score (Mean ±SD)	38.84 ± 1.35	47.98 ± 2.92	86.82 ± 3.62	58.83 ± 3.39	41.04 ± 4.96
SEM	0.19	0.41	0.36	0.48	0.70
MDC_95_	0.53	1.15	1.00	1.33	1.94
Δ (Discharged minus 3-months)	4.23	3.61	5.89	7.67	1.42
SRM	0.81	0.37	0.81	1.67	0.50
ES	1.39	0.78	1.16	1.45	0.10

Abbreviations: ADL, Activities of Daily Living; PCS, Physical Component Summary; MCS, Mental Component Summary; SEM, Standard Error of Measurement; MDC95, Minimal Detectable Change at 95% Confidence Interval; Δ, Change; 3-months, 3 months after discharge; SRM, Standardized Response Mean; Effect Size, ES.

## Supplementary Materials

The following are available online at https://www.mdpi.com/2075-4418/10/3/169/s1.
