# Supplementary material for: Cross-Cultural Adaptation of the Dance Functional Outcome Survey (DFOS) for Spanish Dancers
Source: Diagnostics (Basel). 2020 Mar 20;10(3):169. doi: 10.3390/diagnostics10030169 (PMC7151173; doi:10.3390/diagnostics10030169)
Supplement: Supplementary file 1 [file diagnostics-10-00169-s001.pdf]

Appendix A: Spanish version (DFOS-Sp) of the Dance Functional Outcome Survey

Nombre: ______________________________ Fecha: ______________________________

Por favor, responda a las preguntas de cada sección, eligiendo la opción que mejor la describa. Entendemos que puede que haya más de una opción que la describa, pero señale solo una: la que mejor describa su situación actual. Estas preguntas se basan exclusivamente en lo que puede hacer en este momento. No se compare con otros bailarines. Si una sección no es aplicable, sáltela.

A. RENDIMIENTO GENERAL

1. Nivel de Actividad General

__ No tengo limitaciones. Puedo hacerlo todo, incluso ejercicios y baile extenuante.
__ Puedo bailar, pero a un nivel inferior. Debo guardarme y limitar la cantidad de baile intenso.
__ Puedo realizar baile ligero con problemas ocasionales.
__ No puedo bailar. Las actividades diarias son posibles con problemas ocasionales.
__ Las actividades diarias me provocan problemas moderados
__ Las actividades diarias me provocan problemas severos.

2. Calidad de Movimiento

__ Me siento seguro/a de que puedo rendir al mismo nivel y misma calidad como antes de mi lesión. Soy capaz de articular mis extremidades al 100% de seguridad o claridad.

__ Me siento seguro/a pero estoy al mismo nivel y misma calidad de mi rendimiento como antes de mi lesión. Soy capaz de articular mis extremidades al 80% de seguridad o claridad.

__ Estoy mejorando pero tengo dudas de estar al mismo nivel y misma calidad de rendimiento que antes de mi lesión. Soy capaz de articular mis extremidades al 60% de seguridad o claridad.

__ Estoy mejorando pero solo puedo controlar la calidad de movimiento alguna vez. Soy capaz de articular mis extremidades al 40% de seguridad o claridad.

__ Estoy mejorando pero solo comienzo a centrarme en la calidad de movimiento. Soy capaz de articular mis extremidades al 20% de seguridad o claridad.

__ Estoy mejorando pero solo realizo gestos básicos y no soy capaz de centrarme en la calidad del movimiento en este momento.

3. Caminar

__ Normal y sin limitaciones, incluyendo pendientes.
__ Problemas muy leves, distancias relativamente sin límite.
__ Problemas leves, en la mayoría de superficies, hasta 750 metros.
__ Problemas moderados, superficies planas, un máximo de 300 metros.
__ Problemas severos, solo 200 metros.
__ Problemas severos, necesito bastón o muletas.

4. Escaleras

__ Normal, sin límite, subo y bajo escaleras.
__ Problemas muy leves, necesito tener cuidado (rodear uno) subiendo/bajando escaleras.
__ Problemas leves, tengo que ir despacio, sobretodo (rodear uno) subiendo/bajando escaleras.
__ Problemas moderados, solo son posibles 10-15 escalones, particularmente (rodear uno) subiendo/bajando escaleras.
__ Problemas severos, necesito apoyarme en una barandilla, sobretodo (rodear uno) subiendo/bajando escaleras.
__ Problemas severos, solo entre 0-5 escalone con apoyo, sobretodo (rodear uno) subiendo/bajando escaleras.

5. Estabilidad y Síntomas

__ Puedo hacerlo todo sin síntomas de: fallo, bloqueo, agarrotamiento, rechineo o debilidad.
__ Solo tengo síntomas de (fallo, bloqueo, agarrotamiento, rechineo o debilidad) con baile o ejercicio extenuantes.
__ Solo tengo síntomas de (fallo, bloqueo, agarrotamiento, rechineo o debilidad) con baile moderado; tengo limitaciones con actividades vigorosas.
__ Como tengo síntomas de (fallo, bloqueo, agarrotamiento, rechineo o debilidad) con baile ligero, tengo limitaciones en casi todo lo que bailo. Ocasionalmente tengo síntomas al caminar o con tareas domésticas ligeras.
__ Tengo síntomas con frecuencia al realizar actividades sencillas como caminar. Tengo que proteger la lesión en todo momento.
__ Tengo problemas severos con síntomas de (de fallo, bloqueo, agarrotamiento, rechineo o debilidad). No puedo hacer casi nada sin tener síntomas.

6. Dolor

__ No tengo dolor.
__ Tengo dolor ocasional cuando realizo actividad o baile extenuante. No creo que haya vuelto completamente a la normalidad. Las limitaciones son leves y tolerables, si tengo cuidado.
__ Tengo dolor ocasional cuando realizo actividad ligera o baile moderado.
__ Tengo dolor con cualquier baile, ejercicio o actividad ligera. Las actividades diarias provocan dolor ocasional.
__ El dolor es un problema significante incluso con actividades tan sencillas como caminar. El dolor se alivia con el descanso. No puedo realizar ni baile ni ejercicio.
__ Me duele en todo momento, incluso al andar, permanecer de pie o realizar trabajos domésticos ligeros.

B. TECNICA-ESPECIFICA

7. Plié

__ Soy capaz de realizar Grand Plié en todas las posiciones, incluyendo 4° y 5°.

__ Soy capaz de realizar Grand Plié solo en 1° y 2°.

__ Soy capaz de realizar Grand Plié en 2° solamente.

__ No puedo realizar Grand Plié pero puedo hacer Demi Plié en todas las posiciones.

__ Tengo algunas dificultades para realizar el Demi Plié.

__ No puedo realizar el Demi Plié.

8. Développé

__ Soy capaz de realizar completamente todas las partes del Développé hacia adelante o lateral sin problema.

__ Tengo problemas muy leves para realizar el Développé hacia delante o lateral.

__ Tengo problemas leves para extender completamente la pierna en el Développé hacia delante o hacia el lado, y tengo que realizar el Développé a una altura inferior.

__ Tengo problemas moderados para extender completamente la pierna hacia delante o hacia el lado, y debo marcarlo, pero puedo realizar completamente el Passé.

__ No puedo hacer el Développé ni hacia delante ni hacia el lado pero puedo hacer el Passé completo.

__ No puedo realizar completamente el Passé.

9. Relevé Balance

(Si trabajas en Pointe (Puntas), indica si puedes realizar el nivel indicado en Pointe).

__ Soy capaz de alcanzar y mantener el equilibrio en Relevé/Pointe en el lado de la lesión sin problemas.

__ Soy capaz de alcanzar y mantener el equilibrio en Relevé/Pointe en el lado de la lesión con problemas leves.

__ Soy capaz de alcanzar y mantener el equilibrio en Relevé/Pointe en el lado de la lesión con moderado problema.

__ Soy capaz de realizar Releve pero no puedo mantener el equilibrio en el lado de la lesión sin ayuda de una barra.

__ Soy capaz de mantener el equilibrio con el pie apoyado en el suelo pero no en Relevé.

__ No puedo hace Relevé ni mantener el equilibrio con el lado de la lesión en el suelo.

10. Rond de Jambe

__ Soy capaz de realizar tanto y con tanta frecuencia como sea necesaria, a 90°, Grande Rond de Jambe en l'aire a la Seconde (movimientos rotacionales de la pierna en el aire).

__ Soy capaz de realizar a velocidad reducida: Rond de Jambe en l'aire a la seconde (movimientos rotacionales de la pierna en el aire).
__ Soy capaz de realizar con leves problemas tales como cantidad y velocidad reducida: Rond de Jambe en l'aire a la Seconde (movimientos rotacionales de la pierna en el aire).
__ Puedo realizar con problema moderado tales como cantidad, velocidad y altura reducidos (45°): Rond de Jambe en l'aire a la Seconde (movimientos rotacionales de la pierna en el aire)
__ Marco o evito todo movimiento tipo Rond de Jambe en l'aire (movimientos rotacionales de la pierna en el aire).
__ No puedo realizar Rond de Jambe en l'aire a la Seconde (movimientos rotacionales de la pierna en el aire).

11. Arrodillarse/Trabajos de suelo

__ Soy capaz de realizar trabajo de suelo y arrodillarme, sin limitaciones.
__ Soy capaz de realizar trabajo de suelo y arrodillarme, con limitaciones muy leves.
__ Soy capaz de realizar trabajo de suelo y arrodillarme, con limitaciones moderadas.
__ Soy capaz de realizar trabajo de suelo y arrodillarme, con más limitaciones moderadas; requiere menos repeticiones o ligeras modificaciones.
__ Problemas graves, necesito apoyo o modificación.
__ Problemas graves, no lo puedo realizar.

12. Giros

__ Soy capaz de realizar completamente giros múltiples de todo tipo, en cada pierna (igual que antes de la lesión)
__ Soy capaz de realizar, pero no completamente, giros de todo tipo, en cualquier pierna (igual que antes de la lesión).
__ Soy capaz de realizar la mayoría de giros con ligero problema, en cualquier pierna. Debo tener cuidado con la colocación.
__ Tengo dificultad moderada con los giros. Solo puedo hacer un giro sencillo hacia dentro y hacia fuera en el lado de la lesión.
__ Tengo problemas severos, no puedo hacer giros. Solo puedo hacer la preparación del giro y el equilibrio en Releve en el lado de la lesión.
__ Tengo problemas graves, no puedo mantener el equilibrio en el lado de la lesión.

13. Saltos

__ Soy capaz de realizar todo plenamente: todas las combinaciones de Grand y Petit Allegro (saltos grandes y pequeños), incluyendo tiempo musical (al mismo nivel que antes de la lesión). La potencia de salto es normal y sin límite. Puedo mantener el equilibrio al aterrizar de un salto.
__ Soy capaz de realizar, aunque no del todo completamente (al mismo nivel que antes de la lesión). La potencia de salto y la capacidad de mantener el equilibrio al aterrizar son bastante buenas.

__ Soy capaz de realizar, pero con leves problemas y una ligera protección: Grande y Petit Allegro, y mantener el equilibrio al aterrizar de saltos. Evito la mayoría de saltos difíciles. No puedo hacer saltos repetidos.
__ Tengo problemas moderados con los saltos. Solo puedo hacer saltos sencillos en el centro.
__ Problemas severos, que afectan a todos los saltos en el centro. Puedo hacer saltos simples en la barra.
__ Problemas severos, no puedo saltar.

14. Grand Allegro / A través suelo / Vuelo / Correr

__ Soy capaz de realizar todas las combinaciones de ejercicios de desplazamiento en el espacio (cambio de dirección, pivotar, arranques y paradas en seco, correr) a plena velocidad.
__ Soy capaz de realizar, pero no del todo, todas las combinaciones de ejercicios de desplazamiento en el espacio (cambio de dirección, pivotar, arranques y paradas en seco, correr)
__ Soy capaz de realizar, con problemas leves, todas las combinaciones de ejercicios de desplazamiento en el espacio (cambio de dirección, pivotar, arranques y paradas en seco, correr) a velocidad reducida.
__ Tengo moderado problema y debo moverme despacio y con precaución en las combinaciones de ejercicios de desplazamiento en el espacio (cambio de dirección, pivotar, arranques y paradas en seco, correr).
__ Tengo problemas severos y debo evitar la mayoría de las combinaciones de ejercicios de desplazamiento en el espacio. Me limito a la barra y lentamente (o centro del suelo).
__ Evito todas las combinaciones de de ejercicios de desplazamiento en el espacio.

Como bailarín/a sano/a, o comparándolo con antes de mi lesión, si tuviera que puntuar mi rendimiento como bailarín/a en una escala de 0 a 100, donde 0 es la peor y 100 la mejor, me daría un valor de ___________.

Appendix B: DFOS-Sp Scoring

A. Rendimiento General

1. Nivel de Actividad General	10
2. Calidad de Movimiento	10
3. Caminar	5
4. Escaleras	5
5. Estabilidad y Síntomas	5
6. Dolor	5

Subtotal = 40

B. Tecnica-Especifica

7. Plié	5
8. Développé	5
9. Relevé Balance	5
10. Rond de jambe	5
11. Arrodillarse /Trabajos de suelo	5
12. Giros	5
13. Saltos	10
14. Grand Allegro / A través suelo / Vuelo / Correr	10

Subtotal = 50

Total = 90

Total puntaje = % [e.g. 90/90 = 100%]

Si una pregunta no es respondida, tome una proporción de las preguntas contestadas.

Última pregunta: Como bailarín/a sano/a, o comparándolo con antes de mi lesión, si tuviera que puntuar mi rendimiento como bailarín/a en una escala de 0 a 100, donde 0 es la peor y 100 la mejor, me daría un valor de ___________.

Porque esta pregunta es sobre 100, it is a percentage. es un porcentaje.
